# Spatio-temporal epidemiology of hand, foot and mouth disease in Liaocheng City, North China

**DOI:** 10.3892/etm.2015.2207

**Published:** 2015-01-22

**Authors:** SHIYING ZHANG, JINXING ZHAO

**Affiliations:** Department of Communicable Disease Control and Prevention, Center for Disease Control and Prevention of Liaocheng City, Liaocheng, Shandong 252000, P.R. China

**Keywords:** epidemiology, foot-and-mouth disease, enterovirus 71, disease control and prevention

## Abstract

Hand, foot and mouth disease (HFMD) has posed a notable threat to public health and become a public health priority in China. This study was based on the reported cases of HFMD between 2007 and 2011. A total of 34,176 HFMD cases were geo-coded at town level (n=134). Firstly, a descriptive analysis was conducted to evaluate the epidemic characteristics of HFMD. Then, the Kulldorff scan statistic based on a discrete Poisson model was used to detect spatial-temporal clusters. Spatial distribution of HFMD in Liaocheng City, China from 2007 to 2011 was mapped at town level in the aspects of crude incidence, excess hazard and spatial smoothed incidence. The spatial distribution of HFMD was non-random and clustered with a significant Moran’s I value every year. The local Moran’s I Z-score detected three significant spatial clusters for high incidence of HFMD. The space-time analysis identified one most likely cluster and twenty-five secondary clusters for high incidence of HFMD. We demonstrate evidence of the existence of statistically significant HFMD clusters in Liaocheng City. Our results provide better guidance for formulating regional prevention and control strategies.

## Introduction

Hand, foot and mouth disease (HFMD) is a highly contagious viral disease caused by the foot and mouth disease virus. The most common strains causing HFMD are Coxsackie A virus A16 and enterovirus 71 (EV-71) (1); however, it may be caused by various strains of the Coxsackie virus or enterovirus (2). The disease is characterized by severe vesicular disease in cloven-hoofed animals, including domestic animals and wild species (3). The clinical symptoms usually start with fever, followed by oral vesicles and blisters on the feet. Although these symptoms tend to be self-limiting in adult animals, HFMD causes immense economic loss as the epidemic tends to result in the slaughter of an extremely large number of animals.

In 2008, a large wave of HFMD epidemics occurred in mainland China, Taiwan, Malaysia, Singapore, Hong Kong and other places. In mainland China, the epidemics began in Fuyang City in Anhui province, resulting in 353 severe cases and 22 mortalities, and then rapidly developed into a national-scale epidemic, covering 28 provinces within 3 months with 345,159 reported cases (accounting for 70.59% of the total reported cases in the year) (4,5). The Chinese Ministry of Health has listed HFMD as a notifiable class-C communicable disease since May 2008 (6,7). According to the national network’s surveillance data, a total of 5,031,044 cases were officially reported in China between May 2008 and December 2011.

The magnitude of HFMD epidemics varies in response to the scale of the original infection challenge, the geographical distribution of the animal population at risk and the effectiveness of control efforts during eradication. In dynamic outbreak situations (where, as a result of pre-emptive culling, the animal population at risk is constantly changing, and control measures vary in their effectiveness and intensity of application), the spatial and temporal components of disease risk change markedly throughout an epidemic’s course.

In recent years, the HFMD epidemic has rapidly spread in Liaocheng City, China. The incident cases have increased year by year and caused serious social harm. Therefore, in this study we analyzed the prevalence of hand, foot and mouth disease characteristics and trends in Liaocheng City from 2007 to 2011 and study the epidemic strain. We analyzed risk factors in several publications of HFMD, explore prevention and control measures, preliminarily evaluate the effect of prevention and control, and provide a scientific basis for prevention and control work in the future.

## Materials and methods

### Study area and period

Liaocheng is a prefecture-level city in western Shandong province, China. Its geographical location is 36.27N and 115.59E, and it has a population of 5.79 million (data from 2011 census). The Round-Robin Database occupies a land area of 8,715 km^2^. It borders the provincial capital of Jinan to the southeast, Dezhou to the northeast, Tai’an to the south, and the provinces of Hebei and Henan to the west.

The city of Liaocheng administers eight county-level divisions, including one district, one county-level city and six counties: Dongchangfu district, Linqing City, Yanggu County, Dong’e County, Chiping County, Gaotang County, Guan County and Shen County. These are further divided into 134 township-level divisions.

The period of interest was from January 1, 2007 to December 31, 2011. Data relating to HFMD cases were obtained from the National Center for Public Health Surveillance and Information Services, China Center for Disease Control and Prevention. The date recorded was the date of symptom onset, and every district was required to report HFMD cases via the web-based surveillance system in a unified format, with information including the name, gender, age and address of patients.

### Geographical analyses

For the geographical analysis, techniques available through the Geographical Information System (GIS) were employed. To conduct a GIS-based analysis on the spatial distribution of HFMD, a town-level polygon map at a scale of 1:100,000 was obtained, on which the town-level point layer containing information with regard to latitudes and longitudes of central points of each county was created. Geographical data was used from digital maps from the National Fundamental Geographic Information System, China (http://nfgis.nsdi.gov.cn). All HFMD cases were geo-coded and matched to the town-level polygon and point layers by the administrative code using the software ArcGIS9.3 (ESRI Inc., Redlands, CA, USA).

To assess the risk of HFMD in each town, an excess hazard map was produced. The excess hazard represents the ratio of the observed incidence in each town over the average incidence of all areas; the latter is calculated by the number of cases over the total number of people at risk instead of the annualized incidence of a town (8). Mapping raw estimates of disease occurrence can lead to spurious spatial features. To overcome this problem spatial empirical Bayes smoothing was implemented using SpaceStat software (http://www.biomedware.com/?module=Page&sID=spacestat).

### Spatial autocorrelation analysis

Spatial autocorrelation analyses were performed using SpaceStat. Global Moran’s I statistics were used to discern spatial autocorrelation and detect the spatial distribution pattern of HFMD in Liaocheng City. Local Moran’s I statistics were used to examine the local level of spatial autocorrelation and determine locations of clusters or hotspots. A calculated value of local Moran’s I Z-score (LMiZScore) ≥1.96 indicated that the town and its neighboring towns had a HFMD incidence rate statistically significantly higher than other towns. The number of permutations was set to 999 and P<0.05 was considered to indicate a statistically significant difference.

### Space-time scan statistic

The spatial scan statistic developed by Kulldorff (9) implemented in a software program, SaTScan™ version 9.1 (http://www.satscan.org/), was used to test the presence of statistically significant spatial as well as space-time clusters of HFMD and to identify their approximate locations. The method is defined by a cylindrical window with a circular geographic base and with height corresponding to time (10). The null hypothesis assumed that the relative risk (RR) of HFMD was the same within the window compared with outside.

For this analysis, a Poisson-based model was used, where the number of events in an area is Poisson distributed according to a known underlying population at risk (11). The geographical size of the window was limited to half the expected number of cases and the length of time was limited to half the total time period (10). The test of significance of the identified clusters was based on comparing the likelihood ratio test statistics against a null distribution obtained from Monte Carlo simulation (12). The number of permutations was set to 999 and P<0.05 was considered to indicate a statistically significant difference.

## Results

### Descriptive analysis of HFMD

**Figure 1:**
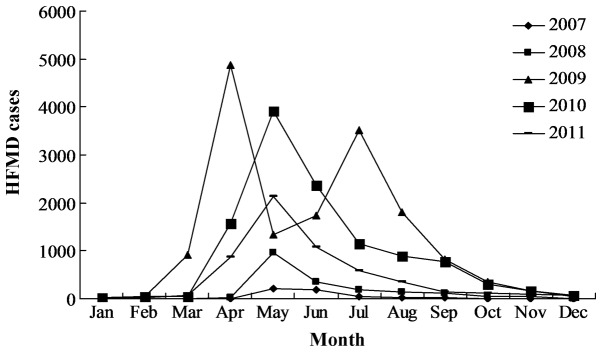
Monthly reported cases of hand, foot and mouth disease (HFMD) in Liaocheng City, China from 2007–2011.

Between January 1, 2007 and December 31, 2011, there were a total of 35,163 HFMD cases reported in Liaocheng City. Of these, 34,176 (98.73%) had complete information, including mapping of their place of residence. Annualized average incidence at the town-level ranged from 2.53% in 2007 to 5.69% in 2009. During the five-year study period, a summer peak was observed in April and June with a second smaller peak in July and August, with the exception of the year 2009, when the peak appearing in April happened to coincide with the influenza (H1N1) pandemic period (Fig. 1).

**Figure 2:**
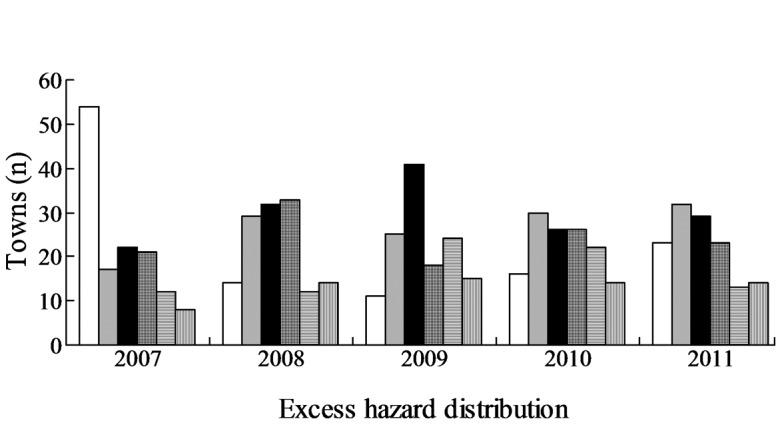
Excess hazard map of hand, foot and mouth disease in Liaocheng City, China from 2007 to 2011.

The excess hazard map reveals the distribution of the excess risk, which was defined as a ratio of the observed number over the expected number of cases. Towns with an excess hazard distribution <0.25 had lower incidences than expected, as indicated by excess risk values <1. In contrast, towns with an excess hazard distribution >0.4 had higher incidences than expected or excess risk values >1 (Fig. 2).

A spatial empirical Bayes smoothed map for annualized average incidence was created by correcting the variance in the variability of incidence (data not shown). The resulting smoothed regional estimates demonstrate a variance stabilizing side effect by using from local or global neighborhood information.

### Spatial autocorrelation analysis of HFMD

The global spatial autocorrelation analyses for annualized incidence of HFMD in Liaocheng City from 2007 to 2011 demonstrated that the Moran’s I value was significant for every year (Table I), implying that the distribution of HFMD was spatially autocorrelated in Liaocheng City, China.

**Figure 3:**
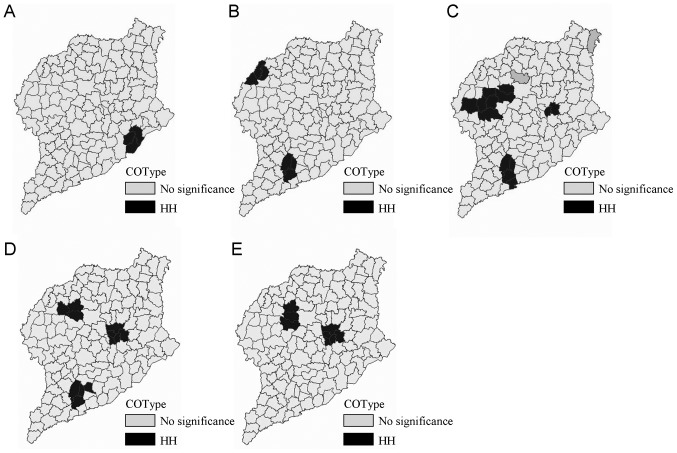
Spatial clustering of hand, foot and mouth disease identified in Liaocheng City, China in 2007 (A), 2008 (B), 2009 (C), 2010 (D) and 2011 (E). COType, congregate; HH, high congregate hotspot.

Three significant spatial clusters of HFMD were identified using the LMiZScore for spatial autocorrelation (Fig. 3). The hotspots persisted in Dongchangfu district, Guan county and Yanggu county from 2007 to 2011.

### Space-time analysis of HFMD

The space-time cluster analysis of HFMD from 2007 to 2011 revealed that HFMD was not distributed randomly in space-time. The most likely statistically significant cluster for high incidence of HFMD was found to exist in Dongchangfu district for the year 2008 (RR=12.83, P<0.01), with 520 observed cases and 235.93 expected cases.

**Figure 4:**
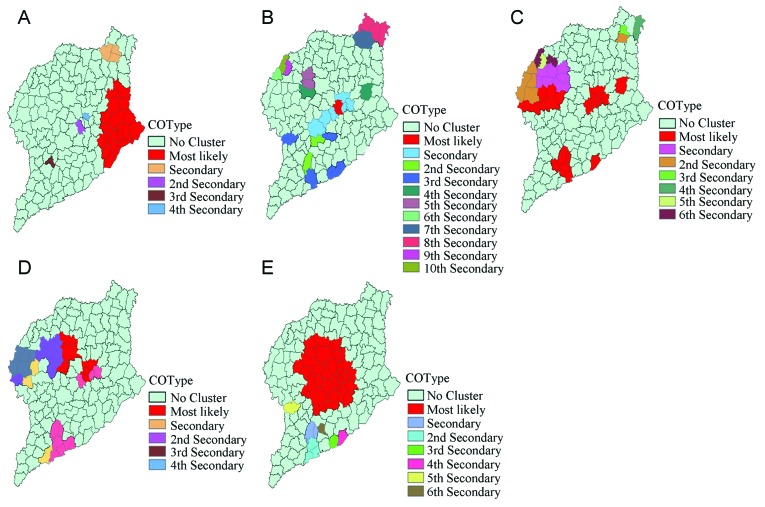
Spatial-time distribution of the identified clusters of hand, foot and mouth disease cases with significant high incidences in Liaocheng City, China in 2007 (A), 2008 (B), 2009 (C), 2010 (D) and 2011 (E). COType, congregate.

Twenty-five statistically significant secondary clusters were also detected for high incidence of HFMD. These results are listed in Table II, and are also depicted on the map in Fig. 4.

## Discussion

Cluster analyses are essential in epidemiology in order to detect aggregation of disease cases, and to test the occurrence of any statistically significant clusters. Cluster analysis identifies whether geographically grouped cases of disease can be explained by chance or are statistically significant. It detects true clusters of disease from cases grouped around population centers. The use of GIS with spatial statistics including spatial filtering and cluster analysis has been applied to a number of diseases to analyze and more clearly demonstrate the spatial patterns of these diseases (13–18). Spatial scan statistics (19) implemented in SaTScan software are being widely used to detect clusters of various diseases worldwide (10,19–29). The results of our space-time analyses clearly demonstrate that the HFMD outbreaks were clustered in space and time in Liaocheng City during the four years studied. The study revealed that the spatial distribution of HFMD in Liaocheng City was non-random and clustered with a significant Moran’s I value every year. LMiZScore detected 25 significant spatial clusters for high incidence of HFMD when only space distribution was considered.

The results of the present study provide useful information on the prevailing epidemiological situation of HFMD in Liaocheng City. This new knowledge of the presence of clusters of HFMD in Liaocheng City may help the Liaocheng Institute to intensify their remedial measures in the identified areas of high HFMD prevalence and determine future strategies for more effective HFMD control. The district health authorities should put more focus into controlling the spread of HFMD in the district. In particular, vigorous efforts are required to intensify case-finding activities in these three HFMD-infested areas of the district. Compulsory HFMD immunization of children, increased coordination between the government and private sector, further promotion of general health and hygiene, and improvement in the nutritional status of the towns analyzed in the study may lead to better control of HFMD in the district.

The present study only analyzes the statistically significant clusters of HFMD in Liaocheng City. Future research could focus on the effect of various socio-economic and environmental factors on the high occurrence of HFMD. It is an established fact that the incidence of HFMD increases with age. There are several other risk factors responsible for the disease, including malnutrition. This adversely affects the immune system, and may therefore enhance HFMD incidence. The factors mainly responsible for the high occurrence of HFMD may be attributable to the poor socio-economic conditions of the inhabitants and poor nutrition. Now that the statistically significant clusters of HFMD have been identified in the region, a survey-based study is planned to identify the role of these factors in the spread of HFMD.

The present study has revealed the presence of three hotspots of HFMD in Liaocheng City, China. Spatial statistics and GIS may provide public health officials with necessary feedback on the prevalence of statistically significant clusters of HFMD in the region, and thus enable them to develop more effective strategies to control HFMD. Since the efficacy of HFMD control measures in specific areas could be assessed by a longitudinal change in HFMD prevalence, the space-time scan statistics also may contribute to a health program evaluation. More detailed individual-level investigations are needed in the identified clusters to evaluate the most significant determinants of disease distribution.

## Figures and Tables

**Table I:** Global spatial autocorrelation analyses for annualized incidence of hand, foot and mouth disease in Liaocheng City from 2007 to 2011.

Year	Moran’s *I*	E (I)	SD	P-value	Pattern
2007	0.1440	−0.0075	0.0013	0.000026	Clustered
2008	0.3316	−0.0075	0.0029	0.000000	Clustered
2009	0.5878	−0.0075	0.0038	0.000000	Clustered
2010	0.5263	−0.0075	0.0035	0.000000	Clustered
2011	0.5903	−0.0075	0.0036	0.000000	Clustered

**Table II:** SaTScan statistics for space-time clusters with significant higher incidence in Liaocheng City, China from 2007 to 2011.

Time	Cluster type	Cluster areas (n)	Observed cases	Expected cases	Relative risk	P-value
2007	Most likely	17	206	52.18	6.49	<0.01
2007	Secondary	3	32	8.38	4.04	<0.01
2008	Most likely	3	85	6.91	12.83	<0.01
2008	Secondary	6	193	61.26	3.40	<0.01
2008	2nd secondary	3	58	9.80	6.08	<0.01
2008	3rd secondary	7	223	108.40	2.20	<0.01
2008	4th secondary	2	33	6.72	4.98	<0.01
2008	5th secondary	2	32	8.36	3.88	<0.01
2008	6th secondary	1	27	6.31	4.33	<0.01
2008	7th secondary	3	77	40.11	1.96	<0.01
2008	8th secondary	3	34	12.78	2.69	<0.01
2009	Most likely	20	2668	912.45	3.33	<0.01
2009	Secondary	7	2003	866.30	2.51	<0.01
2009	2nd secondary	5	750	193.30	4.03	<0.01
2009	3rd secondary	1	137	33.44	4.13	<0.01
2009	4th secondary	1	356	182.97	1.97	<0.01
2009	5th secondary	1	55	8.35	6.61	<0.01
2009	6th secondary	2	242	167.53	1.45	<0.01
2010	Most likely	7	2117	604.05	4.10	<0.01
2010	Secondary	9	1262	407.26	3.37	<0.01
2010	2nd secondary	3	344	57.39	6.16	<0.01
2010	3rd secondary	7	477	241.84	2.02	<0.01
2011	Most likely	31	2964	1230.19	4.08	<0.01
2011	Secondary	2	69	15.60	4.47	<0.01
2011	2nd secondary	3	52	21.29	2.46	<0.01
